# Genomic Instability in Circulating Tumor Cells

**DOI:** 10.3390/cancers12103001

**Published:** 2020-10-16

**Authors:** Monique Oliveira Freitas, John Gartner, Aline Rangel-Pozzo, Sabine Mai

**Affiliations:** 1 Cell Biology, Research Institute of Oncology and Hematology, University of Manitoba, Cancer Care Manitoba, Winnipeg, MB R3C 2B7, Canada; niquecullen@gmail.com; 2 Genetic Service, Institute of Paediatrics and Puericulture Martagão Gesteira (IPPMG), Federal University of Rio de Janeiro (UFRJ), Rio de Janeiro 21941-912, Brazil; 3 Clinical Medicine Postgraduate Programme, College of Medicine, Federal University of Rio de Janeiro (UFRJ), Rio de Janeiro 21941-913, Brazil; 4 Departments of Pathology and Immunology, Faculty of Health Sciences, University of Manitoba, Winnipeg, MB R3E 3P5, Canada; john.gartner@umanitoba.ca

**Keywords:** circulating tumor cells (CTCs), chromosome instability, tumor heterogeneity

## Abstract

**Simple Summary:**

In this review, we focus on recent advances in the detection and quantification of tumor cell heterogeneity and genomic instability of CTCs and the contribution of chromosome instability studies to genetic heterogeneity in CTCs at the single-CTC level.

**Abstract:**

Circulating tumor cells (CTCs) can promote distant metastases and can be obtained through minimally invasive liquid biopsy for clinical assessment in cancer patients. Having both genomic heterogeneity and instability as common features, the genetic characterization of CTCs can serve as a powerful tool for a better understanding of the molecular changes occurring at tumor initiation and during tumor progression/metastasis. In this review, we will highlight recent advances in the detection and quantification of tumor cell heterogeneity and genomic instability in CTCs. We will focus on the contribution of chromosome instability studies to genetic heterogeneity in CTCs at the single-CTC level by discussing data from different cancer subtypes and their impact on diagnosis and precision medicine.

## 1. Introduction

Tumor metastasis, a major causative event involved in cancer mortality, is preceded by the appearance of circulating tumor cells (CTCs) in the blood. CTCs can be obtained through a minimally invasive liquid biopsy regardless of whether they are derived from the primary tumor or from metastatic sites. CTCs analyses can provide important information for disease monitoring and contribute to precise and personalized management for cancer patients. Beyond clinical applications, CTCs can be used as predictive biomarkers of tumor progression, patient survival, and metastatic potential. Genetic tumor heterogeneity and genomic instability are common features of CTCs, with chromosomal instability (CIN) being the most common form of genomic instability in cancer cells. The analysis of CIN in CTCs allows for the tracking of the genetic alterations that gave rise to the tumor and contribute to the detection of subpopulations that comprise intratumor heterogeneity. In this review, we will conceptualize chromosomal instability and tumor heterogeneity, discuss methods for CTCs enrichment and isolation, and outline techniques used to assess chromosome instability in CTCs. We will also highlight recent advances in the detection and quantification of tumor cell heterogeneity and genomic instability of CTCs. We will focus on the contribution of chromosome instability studies of CTCs at the single-cell level and discuss data from studies of different types of cancer.

## 2. Circulating Tumor Cells (CTCs)

Tumor metastasis is associated with high mortality in cancer and is preceded by the presence of CTCs in the blood [1]. CTCs are rare cells (1 per 10^6^–10^7^ leukocytes) in the peripheral bloodstream [2,3]. Genetic modifications in CTCs allow these cells to intravasate into blood and lymphatic vessels and to metastasize into other tissues and organs [4]. The first study to describe CTCs in the blood of cancer patients was performed by Ashworth in 1869 [5,6]. His findings raised the possibility that CTCs could be used as a valuable, minimally invasive “liquid biopsy” for clinical staging in cancer patients. Initial studies showed that the presence of CTCs is associated with short survival [6,7,8] and explored the use of CTCs as predictive biomarkers of tumor progression and metastases as well as patient survival [1,6,9,10,11], even in early stages of the disease [12,13]. CTCs analysis can also be implemented to improve the understanding of the genetic changes that occur in primary tumor cells that lead to extravasation and cancer progression [1,4,12].

### CTCs Isolation and Detection Studies

Baccelli et al. (2013), using the CellSearch system, the only clinically validated, FDA-approved system for identification, isolation, and enumeration of CTCs, showed that only 1.43% of breast cancer patients had more than 500 CTCs per 7.5 mL of blood [4,14]. This study also highlighted an important feature of CTCs: these cells have a diameter that is three to four times larger than that of normal blood cells and capillaries [1,9]. This means that only extremely small and/or pliable CTCs can circulate in the blood [4]. Therefore, for the identification and collection of CTCs in reasonable numbers, several innovative detection and enrichment technologies have been developed. Most of these take advantage of CTCs’ physical and biological properties [11]. CTCs can be enriched either positively or negatively based on biological properties such as expression of cell surface protein markers or physical characteristics such as size, density, deformability, or electrical charge [15].

Lianidou and Pantel (2018) reviewed current methodologies for CTC isolation, enumeration, and detection, and addressed clinical applications of CTC analysis in breast and prostate cancers [16]. Regardless of the isolation method used, the detection of these cells is confirmed in some cases by (1) visualization of an intact nucleus using a DNA binding fluorescent stain, DAPI (4′,6-diamidino-2-phenylindole); (2) expression of cytokeratin and other epithelial or specific cell surface markers such as SNAIL and ALDH1 (used to isolate CTCs in colon cancer); and/or (3) absence of expression of a white blood cell marker, such as CD45—the leukocyte-common antigen gene [1,17,18,19].

CTCs have also been detected by the expression of combined epithelial markers such as EPCAM and cytokeratin (CK), which are expressed on normal epithelial cells and carcinomas but are absent on blood leukocytes [20]. These surface markers can be employed to distinguish cancer cells from normal blood cells [21]. The drawback of this approach is known as the epithelial-to-mesenchymal transition (EMT). EMT is a reversible and transitory process that enables a cell with an epithelial phenotype to switch to a mesenchymal or fibroblastoid cellular phenotype [3]. EMT is involved in an increased epithelial tumor cell migration capacity, invasion, dissemination, plasticity, and immune resistance. It enhances the ability of cancer cells to become CTCs and survive in the bloodstream. EMT can also reduce the expression of epithelial markers [21]. Reduced EPCAM expression of some CTCs has prompted the importance of identifying and validating new CTC markers [22,23,24]. Cayrefourcq et al. (2015) found that colon CTCs co-expressed SNAIL, ALDH1, and CD133 with EPCAM and cytokeratin [23]. Baccelli et al. (2013) showed that breast cancer CTCs, xenografted into immunodeficient mice, expressed EPCAM and cytokeratins along with CD44, CD47, and MET oncoprotein [14]. This combination turned out to be a strong indicator of both decreased progression-free survival and overall survival in patients with metastatic breast cancer. Baccelli et al. (2013) also demonstrated substantial interpatient CTC heterogeneity. They found EPCAM-negative and EPCAM low expression in breast cancer CTCs, underlining the weakness of EPCAM-based isolation methods [14]. Furthermore, Dong et al. (2020) found different phenotypic cell surface markers (CK +/EpCAM-, CK-/EpCAM +, CK +/EPCAM +) in CTCs from prostate cancer patients isolated by two different methods that do not depend on cell surface expression markers [25]. They used a selection-free platform known as Rarecyte and a size-based platform named FAST [25]. Rienbensahm et al. (2019) also compared the efficiency of EPCAM isolation and they found that CTCs were detected in 47.7% of the patients after EpCAM-dependent enrichment, in 32.6% after EpCAM-independent enrichment, and in 63.6% with both enrichment methods combined [26].

Ye et al. (2019) analyzed CTCs from 19 different carcinomas in different stages and found that EpCAM expression was more frequent in CTCs than vimentin expression [27]. Vimentin is a member of the intermediate filament family of proteins and a marker of EMT [28]. Interestingly, the expression of vimentin was observed mostly in small CTCs rather than in large ones [27]. Small CTCs are closely associated with EMT and responsible for relapse and cancer metastasis [29]. Comaills et al. (2016) showed that epithelial cells, which failed to undergo proliferation arrest during EMT, presented mitotic abnormalities and aneuploidy (presence of an abnormal number of chromosomes in a cell) [30]. This higher level of genetic instability was correlated with an increased expression of mesenchymal markers [30].

## 3. Genetic and Chromosome Instability

Genetic heterogeneity and genomic instability are common features of CTCs in almost all human cancers [4,31]. Genomic instability refers to an increased tendency of genetic alterations likely to develop during cell division [32]. The accumulation of changes is a driving force for the development, transformation, and progression of tumor cells and results in intratumoral cell heterogeneity [1,32,33]. Small structural variations such as base pair mutation and microsatellite instability (MSI) or significant chromosome structural variations such as changes in chromosome number or structure (duplication, deletion, and translocation) are markers of genomic or chromosomal instability (CIN).

CIN is defined as a high rate of change in the structure and/or number of chromosomes over time [31,34]. It involves gain and/or loss of whole chromosomes or chromosome fragments. It was first described over a century ago and was linked to tumorigenesis [31,35]. Experimental evidence suggests that CIN enables tumor cells to progress and to adapt selectively to different environmental pressures. Aneuploidy, a consequence of CIN, has been implicated in tumorigenesis [35,36,37] based on the increased rates of malignancy found in patients with global or mosaic aneuploidies [38,39,40]. Detection of CIN requires the determination of chromosome mis-segregation rates. Cells derived from CIN precursors show high levels of variation in chromosome content in contrast to cells derived from stable precursors [41]. To measure the rate of change in chromosome number as a measure of chromosomal instability in colorectal cancer cell lines, Lengauer et al. (1997) performed fluorescence in situ hybridization (FISH) with a panel of centromeric probes and microsatellite analysis [42]. The authors discovered that tumor cell lines without microsatellite instability exhibited CIN through defects in chromosome segregation with alterations in excess of 10^−2^ gain or loss per chromosome per generation [42]. This chromosomal instability was shown to be dominant and was confirmed by introducing a single copy of chromosomes in a stable colorectal tumor cell line [42].

A high rate of chromosomal instability in tumor clones has been reported in other malignancies such as breast and lung cancers [43,44]. Increased expression of mitotic checkpoint genes in breast cancer, increased microtubule assembly rates in colorectal cancer, and deviant kinetochore microtubule dynamics have also been reported to contribute to chromosomal instability and cancer development [45,46,47].

Different gene networks are essential to control cell survival and genome integrity in normal cells. Alterations that involve activation or inactivation of any of these key genes can initiate CIN [41]. An important example of a gene involved in the CIN phenotype is *TP53*. Several studies have linked the CIN phenotype with the inactivation or mutation of *TP53* [44,48,49,50,51,52,53]. *TP53* is a tumor-suppressor gene that encodes a nuclear phosphoprotein important in the control of normal cell proliferation, repair of DNA damage, and apoptosis [54]. Genetic alterations in *TP53* are found in 60% of human malignant tumors [41,54,55,56]. Many other proteins have also been associated with CIN, such as APC, BRCA1, Bub3, and EB1, among others [57,58,59,60]. These proteins were summarized by Thompson et al. (2010) along with the possible mechanisms connecting them to the loss of mitotic fidelity in tumor cells and other cell functions [41].

**Figure 1:**
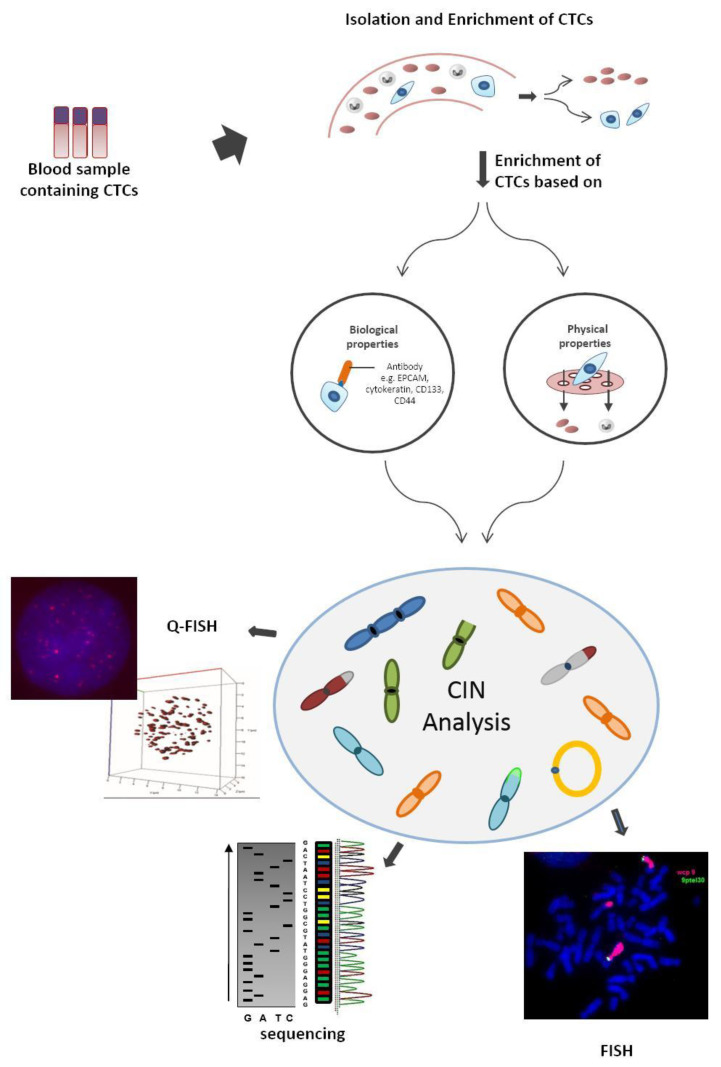
Steps required to obtain circulating tumor cells (CTCs) for chromosomal instability (CIN) analyses and techniques used to characterize chromosome instability. Collection of peripheral blood followed by isolation and enrichment of CTCs based on biological properties (expression of protein markers) or physical properties (size, density, deformability, or electrical charges). After that, CIN analysis can be performed using techniques such as fluorescence in situ hybridization (FISH), whole-exome sequencing, Quantitative fluorescence in situ hybridization (Q-FISH), and next-generation sequencing, among others.

CIN analysis involves the determination of chromosome mis-segregation rates through whole chromosome analysis (FISH with centromeric probes or whole chromosome paints). Analysis of the genes involved in cell cycle control (molecular analysis such as PCR or sequencing for DNA repair genes, mitotic checkpoint genes, etc.) is also used to detect CIN. In all these scenarios, the required tumor cell material is obtained by tumor biopsy—an invasive, costly, and sometimes unfeasible procedure [3], hence the increasing interest in CTC studies. Since CTCs can reflect the chromosomal instability of the primary tumors from which they arise, they allow the identification of relevant biomarkers [3]. This minimally invasive approach can be visualized in Figure 1.

### 3.1. CTCs Data Analysis

In general, CIN analyses are performed using techniques such as FISH, Q-FISH, and next-generation sequencing (analysis of copy number alterations). Recently, CTC platforms such Epic Sciences and RareCyte associated with bioinformatics have allowed the development of different approaches to be used for CTC data analysis in chromosomal instability and genetic heterogeneity [61,62,63,64,65]. Schonhoft et al. (2020) developed a computer vision-based biomarker to detect CIN in CTCs from patients with progressing metastatic castration-resistant prostate cancer (mCRPC) [65]. This image-based algorithm utilizes CTC image features (direct sequencing and morphology) detected by the Epic Sciences platform to predict the presence of a high (nine or more) versus low (eight or fewer) large-scale transitions (LST) number in a single cell [65]. LST are genomic alterations defined as chromosomal breakages of at least 10 Mb of chromosomal gains or losses [65,66,67]. Jendrisak et al. 2020 used the same image-based algorithm to develop a similar CTC-based technology for triple negative breast cancer to identify HRD-like phenotypes [66]. Camptom et al. (2015) [64] characterized the performance of the AccuCyte-CyteFinder system, an integrated technology platform with highly sensitive visual identification and retrieval of individual CTCs from microscopic slides for molecular analysis (after automated immunofluorescence staining for epithelial markers), developed by RareCyte [63,64]. The AccuCyte-CyteFinder provided high-resolution images that allowed the identification of CTCs from prostate, lung, and breast cancer cell lines by morphologic and phenotypic features [64]. Kaldjian et al. (2015) [68] used the same platform, AccuCyte-CyteFinder, to identify CTCs in advanced prostate cancer patients and compare CTC counts with the FDA-cleared CellSearch system (system based on automated immuno-magnetic capture of EpCAM-expressing cells, followed by staining for DNA and cytokeratin to verify that captured cells are nucleated and epithelial in origin) [62,64,68]. The AccuCyte-CyteFinder was able to identify equivalent or greater numbers of CTCs found by the CellSearch system [68]. Aguilar-Avelar et al. (2019) described the design and construction of a fully automated high-throughput fluorescence microscope that enables the recognition, imaging, and classification of CTCs in a blood sample that were labeled by immunostaining [69]. The microscope hardware accurately discriminated CTCs among cells present in blood and the hardware efficiently captured light emitted from unstained cells while the fluorescence signals were used to automatically classify and precisely identify CTCs on a sample by thousands of background cells [69].

### 3.2. Chromosome Instability in CTCs

Manier et al. (2018) compared enriched CTCs with cell-free DNA (cfDNA) [22]. They matched tumor biopsies of 13 patients with multiple myeloma (MM), using whole-exome sequencing (WES), and were able to capture the mutational landscape in the bone marrow, thus providing a comprehensive profile of the clonal heterogeneity and tumor evolution in MM [22]. The combination of CTC analysis and cfDNA was able to detect almost all of the clonal mutations identified in the bone marrow myeloma cells. Manier et al. (2018) also identified recurrent mutated genes, such as *KRAS*, *NRAS*, *BRAF*, and *TP53*, as well as pan-cancer mutations, somatic copy number alterations (1p and 13q deletion, gain of 1q and 11q), and even subclones not identified in the bone marrow [22]. Further, they confirmed that CD138 selection is an important, simple, and quick step towards enriching MM CTCs. They also showed the potential of sequential sample analysis of CTCs by WES to screen disease response and progression during therapy and track future clonal evolution in patients with MM. The similarities between biopsies and CTCs’ genetic background are consistent with two previous reports in breast cancer [53,54]. A high degree of similarity was found in copy number variations (gain of 1q and 8q, and losses of 6q, 8p, distal 11q and 17p) among all analyzed tumor samples of breast cancer (primary tumor, metastasis, CTCs, and cfDNA) [70].

Anantharaman et al. (2016) characterized the expression of the programmed death-ligand 1 (PD-L1) protein [71], which, when expressed in cancer cells, is associated with evasion of immune surveillance and eradication [55]. They analyzed CTCs from patients with muscle invasive and metastatic bladder cancer [71]. The cells, identified by immunofluorescence and genetic characterization (FISH), showed a significant number of genomic aberrations, amplifications, and deletions in multiple chromosomes (1, 2, 6, 17, 18, 20, 21, X, and Y). Xu et al. (2017) used negative enriching immunofluorescence and fluorescence in situ hybridization of chromosome 8 (NE-iFISH) in CTCs from pancreatic cancer patients [72]. They sought to identify CTCs, analyze the chromosomal instability of chromosome 8, and verify the correlation between aneuploidy and prognosis. Patients with CTCs < 3 (monosomic or diploid) chromosome 8 had not only an increased one-year survival but also a higher overall survival when compared to patients with CTCs ≥ 3 (triploid, tetraploid or polyploid) [72]. Similar results were found by Liu et al. (2017) in CTCs of pancreatic ductal adenocarcinoma [73] and by Qiu et al. (2018) in CTCs from thyroid cancer [74]. Chromosome 8 aneuploidy was associated with poor response to radioactive iodine (131I) treatment and worse prognosis in thyroid cancer [74].

Zhang et al. (2018) used subtraction enrichment and an immunostaining-fluorescence in situ hybridization (SE-iFISH) automatic testing system to detect and characterize CTCs in nasopharyngeal carcinoma (NPC) [75]. They found that aneuploidy of chromosome 8 in CTCs was related to chemotherapeutic efficacy [75]. In another study using the same technique, Chen et al. (2019) showed that in newly diagnosed esophageal cancer patients, before and after chemotherapy, non-triploid tumors had a significantly greater clinical response when compared to triploid tumors [76]. This suggests that non-triploid tumors are more sensitive to chemotherapy and that this might serve as a marker to predict chemotherapeutic efficacy [76]. Ye et al. (2019) also used (SE-iFISH) in CTCs from 19 different carcinomas [27]. They found that the total number of CTCs, tetraploid chromosome 8, polyploid chromosome 8, and large CTCs were more frequent in patients with advanced stage cancer (III and IV) when compared to stage I or II [27].

Genetic alterations in CTCs are also a common feature in non-small-cell lung cancer (NSCLC). Different studies have identified mutations in oncogenes associated with tumor growth, such as EGFR mutations, *ALK* gene rearrangement, and alterations in the copy number of ROS-1 and rearrangements [77,78,79,80,81]. All these mutations can lead to the development of CIN. NSCLC CTCs also display substantial heterogeneity of genetic rearrangements, accompanied by a high level of CIN. A high level of CIN is related to increased risk of relapse or death in NSCLC patients, as well as to acquired resistance to ALK inhibitor in ALK-rearranged tumors [82,83]. Carter et al. (2017) emphasized the importance of monitoring CIN in CTCs through the classification of chemosensitive versus chemorefractory small-cell lung cancer (SCLC) patients using copy number aberrations (CNAs) [84]. They demonstrated that CTCs could be useful for the identification of drug resistance biomarkers and expanded our knowledge of tumor heterogeneity driven by genomic instability.

Malihi et al. (2020) analyzed single CTCs from 47 patients with and without aggressive variant prostate cancer (AVPC) [85]. Their results indicated that 42.6% of patients had two or more concurrent losses of tumor-suppressor genes (*PTEN*, *RB1*, and *TP53*) in at least one CTC. This finding was associated with poor survival and increased genomic instability. Loss of the tumor-suppressor genes *PTEN*, *RB1*, and *TP53* was correlated with increased androgen receptor (AR) expression, *BRCA2* gene loss, and gains in chromosomal regions where *PTK2*, *MYC*, and *NCOA2* genes are localized [85]. A comparative analysis of copy number alterations in primary tumor tissue and CTCs (from the same patient) was performed by Gao et al. (2017) and showed that copy number alterations affecting *MYC* and *PTEN* genes were present in all CTCs but not in all matched tissue samples [86]. Lim et al. (2019), using single-cell analysis of CTCs, found the following genomic alterations: microsatellite instability in *BAT 25*, *NR21*, and *NR24* genes; CNAs alteration (amplification or deletion) in *HER2*, *AR*, *CDK8*, and *EGFR*; and insertions or deletions at the single-nucleotide level in the genes *TP53*, *BRAF*, *KRAS*, and *PIK3CA* [87]. Such mutations are not described in the Catalogue of Somatic Mutations in Cancer (COSMIC) database [87,88] and are not found in tissue biopsies [88,89]. These specific alterations were suggested to be involved in CTC phenotypes and provide them with intravasation competency, increased migration/motility, enhanced cell–cell interactions, interaction with platelet, and blood immune cells and resistance to therapy. Such alterations are also linked to CTCs subpopulation and cancer cells phenotypes (cells resistance, metastasis, aggressiveness) [87,90,91].

Another way to characterize CIN and tumor heterogeneity is by studying alterations in telomeres. Telomeres are tandem repeated DNA sequences at the end of chromosomes [92]. The nuclei of cancer cells exhibit telomeric aggregates (TAs) and aberrant telomeric clusters that result in altered 3D telomeric organization in the nuclear space [93,94,95]. Telomere dysfunction has been associated with CIN in different cancer types and the degree of telomeric disorganization can be used to predict the aggressiveness and progression of tumors [96,97,98]. The characterization of subpopulations of CTCs in different types of cancer (colon, prostate, breast, and melanoma), using 3D quantitative analysis of telomeric profiles by quantitative-FISH, was first performed by Awe et al. (2013) [99]. Different subpopulations of CTCs were identified based on their 3D telomeric profiles. In addition, the study showed that changes in the telomere architecture appear to be cancer-specific. CTCs from prostate patients, breast cancer, and melanoma tend to have more telomere aggregates, while CTCs from colon and lung cancer have a significant increase in the number of telomeres [99]. Other studies have indicated that telomere signatures can change over time and during treatment, suggesting marked heterogeneity [13,100]. Julius et al. (2014) collected repeated samples within six-month intervals defined as stable, mildly changing, and significantly altered 3D profiles indicative of disease stability versus progression [100]. Most recently, Drachenberg et al. (2019) and Wark et al. (2019) assessed the individual pretreatment risk of progression in intermediate-risk and high-risk prostate cancer patients undergoing radiation and hormone deprivation therapies, respectively [101,102]. They identified disease heterogeneity among a clinically homogeneous group of prostate cancer patients. In both studies, the telomere profiling divided the patients into three subgroups with different risks of aggressive disease, which suggests differences in therapeutic responses [101,102].

### 3.3. Chromosome Instability and Metastasis Development

In addition to whole chromosomal instability and heterogeneity, other genetic modifications may influence the development of tumors and metastasis. These include genetic amplifications, translocations, or DNA repair deficiencies [103,104,105]. The first two involve structural changes in specific genes or chromosomal regions (i.e., *BCR/ABL* fusion in chronic myelogenous leukemia [106]) while DNA repair deficiencies can affect multiple genes in the cell [104]. DNA repair deficiencies can lead to gene sequences modifications, translation error of proteins, activation of oncogenes or inactivation of tumor-suppressor genes [104,105], and consequently tumor evolution and metastasis [103,104].

Circulating tumor cells are tumor-derived pioneers responsible for the metastatic spread of cancer. High levels of genomic instability and an increased frequency of genetic alterations have been described to fuel metastasis development, even without chemotherapy selection pressure [31,107]. Cancer therapeutic approaches that use DNA damage-inducing agents (taxanes, anthracyclines, platinum compounds) induce apoptosis, overloading the DNA damage response machinery. However, a high rate of DNA damage could also enhance the selection for resistant phenotypes with a high mutational burden [108,109,110].

In triple negative breast cancer patients, Witzel et al. (2018) described that the reactivation of HER2 represents a marker for metastatic progression. HER2-positive CTCs that were not hormone receptor-expressing were detected at higher frequency in patients with metastatic disease when compared to early-stage patients [110,111]. Powell et al. (2016) reported that p53-deficiecy promotes tumor growth, increases in tumor cell shedding into the blood, and enhanced metastasis [112]. Riebensahm et al. (2019) identified alterations in notch (gain of NOTCH3) and *PI3K* (gain of PDPK1) pathways by CNA profiles of CTCs, and alterations in cell cycle regulators such as *TP53*, *RB1*, and *CDKN2A*, as well in genes belonging to the PI3K pathway (PTEN, PIK3CA) and chromatin remodeling (ARID1A) by CTC mutations analysis [26].

In prostate cancer, the degree of phenotypic heterogeneity was considered important in metastatic prostate cancer, marked by a multitude of non-canonical subtypes like CK-negative cells, small CTCs, and CTC clusters [113,114]. Importantly, low CTC heterogeneity was associated with better survival with second-line anti-androgen therapy [115,116]. In pretreatment samples, AR copy number gains or point mutations (T878A or L702H) identified in ctDNA were associated with shorter progression-free survival [117]. In addition, the detection of the AR-V7 transcript, a constitutively active AR splice variant lacking the ligand-binding domain, was associated with worse clinical outcome, drug resistance, and disease progression [114,118].

Interestingly, Losi et al. (2005) observed a reduction in intratumoral genetic heterogeneity from early to advanced stages in colorectal adenocarcinomas. They noticed a 60 to 20% reduction in cells with the *K-ras* mutation and 70 to 20% reduction in cells with the p53 mutation in advanced stages [119]. In non-small-cell lung cancer (NSCLC), *EGFR* mutations, *ALK* gene rearrangement, and alterations in the copy number of ROS-1 were related to increased risk of relapse or death in NSCLC patients, as well as to acquired resistance to ALK inhibitor in ALK-rearranged tumors [77,78,79,80,81,82,83].

It is evident that normal cells and primary tumor cells go through genetic changes that allow them to disseminate and metastasize to distant organs. It is also apparent that tumor cells with CIN produce a heterogeneous population of cells that are able to escape control mechanisms and allow them to selectively adapt to new microenvironments and become resistant to treatment [36]. Tumor cell heterogeneity makes disease monitoring difficult and therapeutic targeting challenging. In this regard, the analysis of CIN in CTCs provides the opportunity to track genetic changes similar to those found in biopsy analyses and to unmask disease heterogeneity.

## 4. Tumor and CTCs Heterogeneity

Intratumor heterogeneity refers to a tumor that contains cells with diverse molecular constitutions [3]. The cell-to-cell heterogeneity in such a tumor can be at the morphological, genomic, transcriptional, and/or protein expression level [3]. The presence of tumor heterogeneity is responsible for different proliferation levels, aggressiveness, and tumor progression of individual cells or cell clones within a single tumor. Tumor heterogeneity directly influences the diagnosis, prognosis of patients, and treatment options [3,120].

**Figure 2:**
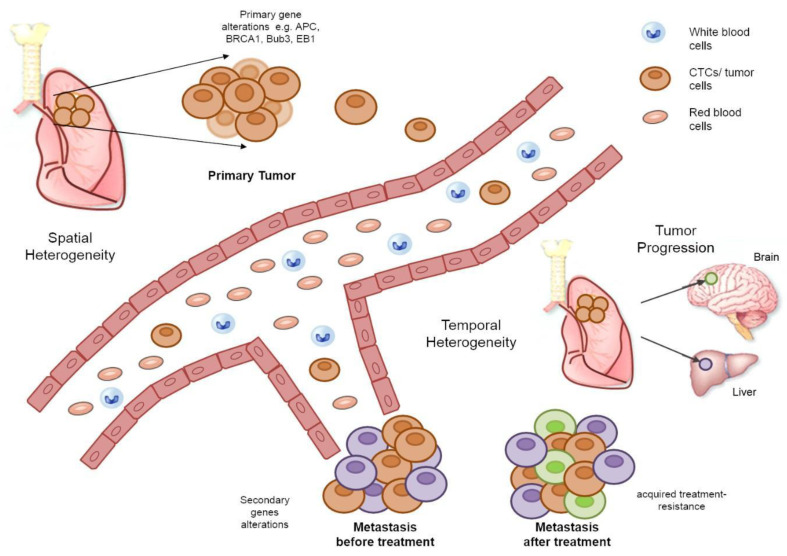
A schematic representation of tumor progression explaining the difference between spatial and temporal tumor heterogeneity. During tumor progression, cells of primary tumor undergo genetic modifications allowing CTCs to intravasate into blood and lymphatic vessels and to disseminate to potential metastatic sites. Tumor cells with CIN produce a heterogeneous population of cells that can escape cell integrity control mechanisms, to adapt to other microenvironments and become resistant to treatments. Spatial heterogeneity reflects differences within a single tumor or between the primary tumor and metastasis. Temporal heterogeneity reflects the emergence of low-frequency tumor subclones and dynamic changes acquired during treatment or secondary gene alterations of tumor cells during disease progression (adapted from Jamal-Hanjani et al. (2015) [126]).

Tumor heterogeneity can be both spatial and temporal [3]. The intra-patient heterogeneity at diagnosis is defined as spatial tumor heterogeneity and reflects differences within a single tumor or between the primary tumor and a metastasis [121,122,123]. Studies designed to monitor tumor progression/evolution and drug resistance mechanisms have revealed the emergence of tumor-resistant subclones [3]. Small changes acquired during disease progression and treatment are referred to as temporal tumor heterogeneity [124,125]. A schematic representation of tumor progression illustrating the difference between spatial and temporal tumor heterogeneity can be seen in Figure 2.

There are two theories to explain tumor heterogeneity. The first one is based on the existence of cancer stem cells. Cancer stem cells are tumor progenitor cells that exhibit unlimited self-renewal capacity coupled with the ability to differentiate [127,128]. The second theory is based on the influence of environmental factors, cell selection, and the Darwinian mode of evolution [129]. Tumor heterogeneity could, therefore, result from those two different but related processes [3,34] in which (1) heterogeneity is a consequence of genetic instability, caused by the accumulation of genetic alterations in cancer stem cells leading to clonal diversity; and (2) heterogeneity is a consequence of differential expression of genes in response to environmental stress. Therefore, studies targeting the characterization of tumor heterogeneity should count on techniques that are able to identify genetic alterations and/or evaluate the expression of important genes for tumor biology and tumor responsiveness.

A large number of technologies have been developed to characterize CIN and genetic heterogeneity in CTCs, including whole genome amplification (WGA), high-throughput or “next-generation sequencing” (NGS), single-cell sequencing (SCS), microarray-based comparative genomic hybridization (array-CGH), and techniques based on FISH, like quantitative FISH (Q-FISH) [3,98,130]. A comprehensive compilation of published studies using CIN to characterize tumor heterogeneity is presented in Table 1.

Heitzer et al. (2013) performed a study using array-CGH and NGS in primary tumors, metastatic lesions, and CTCs of colorectal cancer patients [88]. Among the genes analyzed (68 in total), they identified the genes *KRAS*, *APC*, and *PIK3CA* as commonly mutated between primary tumors, metastatic lesions, and CTCs [88]. Other studies have reported similar results regarding the degree of mutational concordance between tumor tissues (primary tumors and metastasis) and CTCs [131,132,133]. However, some discrepancies have also been observed mainly at the subclonal level [134,135]. The concordance rate of HER-2 expression between CTCs and tumor tissues from breast cancer ranged from 53 to 89% [136,137]. This difference is even higher in non-metastatic breast cancers [138]. Interestingly, CTCs exhibit more heterogeneity, including low-frequency clones, than other samples analyzed, such as cfDNA and biopsy [139]. Indeed, it is still unclear how many CTCs should be analyzed to cover all heterogeneity found in this cell population [140].

Tumor heterogeneity is the main reason for different responses to treatment [139,141]. Lowes et al. (2016) and other studies suggested that tumor heterogeneity is the result of genomic instability along with selective treatment pressure [141,142,143]. This genomic instability results in changes in the genotype of cells, disease progression, and often resistance to treatment [144]. Evidence of low-frequency mutations and subclones prior to or after treatment in patients who acquired treatment resistance has been observed in many types of cancer. For example, epidermal growth factor receptor (EGFR) T790M mutation and MET amplification appear at low frequency prior to treatment (<1% of cells), but are detected at a greater frequency in NSCLC patients CTCs after tyrosine kinase inhibitor (TKI) therapy [145]. This heterogeneity was observed throughout the progression of the disease [146]. Maheswaran et al. (2008) also observed multiple EGFR-activating mutations in CTCs and a trend towards the predominance of specific subclones during treatment [78].

## 5. CTCs Information in Clinical Practice

The ability to assess the mutational changes in real time and over the course of therapy is critically important. In clinical practice, CTCs represent a real-time, minimally invasive alternative source of tumor material. CTCs can be used to assess the landscape of primary and metastatic tumor in the body over time by sampling the blood serially over the course of treatment [147]. CTCs information can be used to monitor disease progression and to predict treatment response. The analysis of CTCs can also be used to select patients for targeted therapies [77,147].

A number of studies have reported the presence of ALK-translocated CTCs in blood from NSCLC patients [80,147,148]. Provencio et al., 2017, used CTCs for the dynamic monitoring of NSCLC patients with ALK rearrangement, as an example [80]. Recently, Pailler et al. (2019) demonstrated that many genes involved in the RTK-*KRAS* and *TP53* pathways were found in patients with crizotinib resistance, showing the clinical utility of CTCs to identify therapeutic resistance mutations in ALK-rearranged patients and for treatment decisions [148].

In prostate cancer, CTC enumeration remains the most extensively validated prognostic marker to date [114], but other clinically relevant phenotypes like androgen receptor (AR) localization or the presence of the AR-V7 splice variant are also in clinical practice. Antonarakis et al. (2014) [149] showed that AR splice variants, in particular AR variant 7 (AR-V7), are strongly associated with primary resistance to abiraterone and enzalutamide therapy in men with CRPC. Recently, another author showed the importance of CTC–based AR-V7 detection as a treatment selection biomarker in CRPC. In AR-V7–positive men, taxanes appear to be more efficient than enzalutamide or abiraterone therapy, while in AR-V7–negative men, taxanes and enzalutamide or abiraterone might have comparable efficacy [118]. Morrison and Goldkorn (2020) summarized the most recent developments for liquid biopsies in advanced prostate cancer with an emphasis on clinical utility for personalized medicine of CTC enumeration, CTC characterization, and next-generation sequencing of CTCs [114].

In breast cancer, Diamantopoulou et al. (2020) evaluated, in a clinical trial, the cardiac glycosides’ effects on CTC clusters in breast cancer patients with progressive disease [150]. Cardiac glycosides are used to dissociate CTC clusters from patient-derived CTCs and prevent spontaneous CTC cluster formation in mouse models [151]. Interestingly, the dissociation of CTC clusters by cardiac glycosides not only resulted in molecular changes that decreased their stem-like traits but also suppressed their direct metastatic ability in preclinical in vivo models [151]. Additional clinical trials have used CTCs’ molecular features such PDL-1 expression in non-small-cell lung cancer and HER2 level in metastatic breast cancer as biomarkers for the identification of high-risk patients [110,150,152].

Clearly, CTCs analysis has important predictive and prognostic value with a strong potential for early cancer diagnosis, screening, and therapeutic decisions. CIN studies using CTCs showed to be fundamental in unmasking disease heterogeneity. However, for the use of CTCs in the clinic, most of the validated biomarkers used for diagnosis and prognosis in tissue biopsies still need to be confirmed [138]. Further, cancer disease heterogeneity represents a challenge for the standardization of dynamic monitoring analysis, adding to the fact that CTCs are present in very low concentration in peripheral blood and this concentration varies according to cancer type and disease stage. For this reason, most studies with CTCs are performed in cancer advanced stages. In some cases, the CTC low detection rate makes necessary the use of other liquid biopsy strategies such as ctDNA as an integrated analysis for results confirmation and to cover the gaps. Lastly, the use of AR-V7 splice variant detection in the clinic for metastatic prostate cancer confirms the value of CTC information and highlights the possibility that many other CTCs biomarkers are just waiting to be uncovered.

## 6. Conclusions

Chromosomal instability and genetic heterogeneity studies are essential for understanding the initiation, evolution, and progression of cancer. Several authors have used CTC analysis for the identification and validation of new cancer markers. They have also used CTCs to track genetic changes acquired during tumor growth and development and to monitor disease progression and resistance to treatment. In this context, CTCs can serve as a surrogate biomarker of the spectrum of molecular characteristics that exist within a tumor. With certain advantages and limitations, CTC analyses can add important predictive and prognostic value.

## Figures and Tables

**Table 1:** Studies of chromosome instability (CIN) analyses that contribute to the characterization of spatial or temporal tumor heterogeneity. Spatial heterogeneity reflects differences within a single tumor or between the primary tumor and metastasis and temporal heterogeneity reflects the presence of low-frequency tumor subclones and dynamic changes in cellular biomarkers of tumor cells during disease progression. CTCs: circulating tumor cells.

Type of Cancer	Author Reference	Evaluated Cells	Techniques	Results and Conclusion	Heterogeneity
Pancreatic cancer	[72]	CTCs	negative enriched immunofluorescence and fluorescence in situ hybridization (NE-iFISH)	chromosomal instability of chromosome 8, and verified correlation between aneuploid CTC and prognosis.	spatial
	[73]	CTCs	fluorescence in situ hybridization (FISH), next-generation sequencing (NGS)	chromosomal instability of chromosome 8, and verified the correlation between aneuploid CTC and prognosis.	spatial
MM	[22]	CTCs, cfDNA, and biopsies	whole-exome sequencing (WES)	identified in CTCs clonal mutations identified in the bone marrow biopsy and even other subclones not identified in the bone marrow	spatial
Breast cancer	[70]	primary tumor, metastasis, CTCs, and cfDNA	whole-exome sequencing (WES), exome, and deep sequencing	high degree of similarities of copy number changes (gain of 1q and 8q, and losses of 6q, 8p, distal 11q and 17p), among all analyzed tumor samples	spatial, temporal
	[15]	metastasis, CTCs and cfDNA	review	similarities in the mutational landscape	spatial, temporal
	[136,137]	CTCs and tumor tissues	fluorescence in situ hybridization (FISH), reverse transcription, and multiplex-PCR	clear heterogeneity in the expression of HER-2 between CTCs and tumor tissues	spatial
	[26]	tumor tissue, brain mestatasis, and CTCs	next-generation sequencing (NGS)	found chromosomal aberrations with a high genomic clonality and mutations in pathways potentially important in brain metastasis formation	spatial
Colon, prostate, breast, and melanoma	[99]	CTCs	3D quantitative analysis of telomeres	characterized subpopulations of CTCs in based on telomere intensity.	spatial
Prostate cancer	[100]	CTCs	3D quantitative analysis of telomeres	characterized genomic instability and heterogeneity in CTCs based in 3D telomeres signatures	spatial
	[101,102]	CTCs	3D quantitative analysis of telomeres	3D telomeres signatures divided the patients into three subgroups with different risks of aggressive disease	temporal
	[140]	CTCs	3D quantitative analysis of telomeres to laser microdissection and single-cell whole-exome sequencing (WES)	characterized genomic instability and heterogeneity based on genetic variation and copy number alteration (CNAs) profiles	spatial
Colorectal cancer	[88]	primary tumors, metastatic lesions, and CTCs	array-CGH and next-generation sequencing (NGS)	profile of the mutation spectrum	spatial
	[131,132,133]	CTCs and tumor tissues	PCR-RFLP or allele-specific PCR, Sanger sequencing, and high-resolution melt	degree of mutational concordance between tumor tissues and CTCs	spatial
Muscle invasive (MIBC) and metastatic (mBCa) bladder cancer	[71]	CTCs	fluorescence in situ hybridization (FISH), next-generation sequencing (NGS)	significant number of genomic aberrations consistent with malignant origin and previous findings of heterogeneity of intratumoral DNA ploidy	spatial
Small-cell lung cancer (SCLC)	[84]	CTCs	analysis of copy number aberrations (CNAs)	classification of chemosensitive versus chemorefractory SCLC patients	spatial, temporal
Non-small-cell lung cancer (NSCLC)	[145]	CTCs and metastases	next-generation sequencing (NGS)	EGFR mutation and MET at a low frequency prior to anti-EGFR treatment, but detected in high frequency in CTCs after TKI therapy or in patients with acquired TKI resistance	temporal
	[78]	CTCs and metastases	allele-specific PCR	multiple EGFR activating mutations in CTCs and a trend towards the predominant evolution of subclones during treatment	temporal
	[81]	CTCs and metastases	filter-adapted-fluorescence in situ hybridization (FA-FISH), DNA content quantification, and chromosome enumeration	alterations in the copy number of ROS-1 rearrangements in CTCs showed temporal heterogeneity during treatment with crizotinib	temporal
	[77,78,79,80,81]	CTCs	multiplex gene-specific PCR and TaqMan allele-specific PCR, fluorescence in situ hybridization (FISH), filter-adapted fluorescence in situ hybridization (FA-FISH)	CTCs display substantial rearrangement heterogeneity accompanied by a high level of CIN	spatial, temporal
Differentiated thyroid cancer (DTC)	[74]	CTCs	negative enriched immunofluorescence and fluorescence in situ hybridization (NE-iFISH)	aneuploidy of chromosome 8 associated with poor response to 131I treatment and worse prognosis	temporal
Nasopharyngeal carcinoma (NPC)	[75]	CTCs	subtraction enrichment and immunostaining-fluorescence in situ hybridization (SE-iFISH)	aneuploidy of chromosome 8 in CTCs was dramatically related to chemotherapeutic efficacy	temporal
Esophageal cancer	[76]	CTCs	subtraction enrichment and immunostaining-fluorescence in situ hybridization (SE-iFISH)	patients with non-triploidy of chromosome 8 are more sensitive to chemotherapy	temporal
19 different carcinomas	[27]	CTCs	subtraction enrichment and immunostaining-fluorescence in situ hybridization (SE-iFISH)	total number of CTCs, tetraploid chromosome 8, polyploid chromosome 8, CTM (Circulating tumor microemboli), and large CTCs were statistically higher in patients with advanced stage cancer	temporal
Aggressive variant prostate cancer (AVPC)	[85]	CTCs	analysis of copy number aberrations (CNAs)	42.6% of patients had concurrent two or more losses of tumor-suppressor genes in at least 1 CTC in association with poor survival and increased genomic instability	Temporal
Colon cancer	[86]	primary tumor and CTCs	analysis of copy number aberrations (CNAs) and single-cell structural variant (SV) analyses	CNAs affecting the MYC gene and the PTEN gene were present in all CTCs, but observed only in some primary tumor cells. Formation of anomalous CNAs in multiple chromosome regions is a result of a complex rearrangement followed by gene amplification	Spatial

## References

[1] Powell A.A., Talasaz A.H., Zhang H., Coram M.A., Reddy A., Deng G., Telli M.L., Advani R.H., Carlson R.W., Mollick J.A. (2012). Single cell profiling of circulating tumor cells: Transcriptional heterogeneity and diversity from breast cancer cell lines. PLoS ONE.

[2] Farace F., Massard C., Vimond N., Drusch F., Jacques N., Billiot F., Laplanche A., Chauchereau A., Lacroix L., Planchard D. (2011). A direct comparison of CellSearch and ISET for circulating tumour-cell detection in patients with metastatic carcinomas. Br. J. Cancer.

[3] Zhang C., Guan Y., Sun Y., Ai D., Guo Q. (2016). Tumor heterogeneity and circulating tumor cells. Cancer Lett..

[4] Plaks V., Koopman C.D., Werb Z. (2013). Circulating tumor cells. Science.

[5] Ashworth T.R. (1869). A case of cancer in which cells similar to those in the tumours were seen in the blood after death. Aust. Med. J..

[6] Cristofanilli M., Budd G.T., Ellis M.J., Stopeck A., Matera J., Miller M.C., Reuben J.M., Doyle G.V., Allard W.J., Terstappen L.W. (2004). Circulating tumor cells, disease progression, and survival in metastatic breast cancer. N. Engl. J. Med..

[7] Seronie-Vivien S., Mery E., Delord J.P., Fillola G., Tkaczuk J., Voigt J.J., Bugat R. (2001). Carcinocythemia as the single extension of breast cancer: Report of a case and review of the literature. Ann. Oncol..

[8] Rodríguez-Salas N., Jiménez-Gordo A.M., González E., Zamora P., Espinosa E., de Castro Fernández M., González-Barón M. (2000). Circulating cancer cells in peripheral blood. A case report. Acta Cytol..

[9] Cohen S.J., Punt C.J., Iannotti N., Saidman B.H., Sabbath K.D., Gabrail N.Y., Picus J., Morse M., Mitchell E., Miller M.C. (2008). Relationship of circulating tumor cells to tumor response, progression-free survival, and overall survival in patients with metastatic colorectal cancer. Clin. Oncol..

[10] De Bono J.S., Scher H.I., Montgomery R.B., Parker C., Miller M.C., Tissing H., Doyle G.V., Terstappen L.W., Pienta K.J., Raghavan D. (2008). Circulating tumor cells predict survival benefit from treatment in metastatic castration-resistant prostate cancer. Clin. Cancer Res..

[11] Alix-Panabières C., Pantel K. (2013). Circulating tumor cells: Liquid biopsy of cancer. Clin. Chem..

[12] Rhim A.D., Mirek E.T., Aiello N.M., Maitra A., Bailey J.M., McAllister F., Reichert M., Beatty G.L., Rustgi A.K., Vonderheide R.H. (2012). EMT and dissemination precede pancreatic tumor formation. Cell.

[13] Wark L., Klonisch T., Awe J., LeClerc C., Dyck B., Quon H., Mai S. (2017). Dynamics of three-dimensional telomere profiles of circulating tumor cells in patients with high-risk prostate cancer who are undergoing androgen deprivation and radiation therapies. Urol. Oncol..

[14] Baccelli I., Schneeweiss A., Riethdorf S., Stenzinger A., Schillert A., Vogel V., Klein C., Saini M., Bäuerle T., Wallwiener M. (2013). Identification of a population of blood circulating tumor cells from breast cancer patients that initiates metastasis in a xenograft assay. Nat. Biotechnol..

[15] Alix-Panabières C., Pantel K. (2016). Clinical applications of circulating tumor cells and circulating tumor DNA as liquid biopsy. Cancer Discov..

[16] Lianidou E., Pantel K. (2019). Liquid biopsies. Genes Chromosomes Cancer.

[17] Allard W.J., Matera J., Miller M.C., Repollet M., Connelly M.C., Rao C., Tibbe A.G., Uhr J.W., Terstappen L.W. (2004). Tumor cells circulate in the peripheral blood of all major carcinomas but not in healthy subjects or patients with nonmalignant diseases. Clin. Cancer Res..

[18] van de Stolpe A., Pantel K., Sleijfer S., Terstappen L.W., Den Toonder J.M. (2011). Circulating Tumor Cell Isolation and Diagnostics: Toward Routine Clinical Use.

[19] Charbonneau H., Tonks N.K., Walsh K.A., Fischer E.H. (1988). The leukocyte common antigen (CD45): A putative receptor-linked protein tyrosine phosphatase. Proc. Natl. Acad. Sci. USA.

[20] Pantel K., Alix-Panabières C. (2013). Real-time liquid biopsy in cancer patients: Fact or fiction?. Cancer Res..

[21] Alix-Panabières C., Pantel K. (2014). Challenges in circulating tumour cell research. Nat. Rev. Cancer.

[22] Manier S., Park J., Capelletti M., Bustoros M., Freeman S.S., Ha G., Rhoades J., Liu C.J., Huynh D., Reed S.C. (2018). Whole-exome sequencing of cell-free DNA and circulating tumor cells in multiple myeloma. Nat. Commun..

[23] Cayrefourcq L., Mazard T., Joosse S., Solassol J., Ramos J., Assenat E., Schumacher U., Costes V., Maudelonde T., Pantel K. (2015). Establishment and characterization of a cell line from human circulating colon cancer cells. Cancer Res..

[24] Paiva B., Paino T., Sayagues J.-M., Garayoa M., San-Segundo L., Martín M., Mota I., Sanchez M.-L., Bárcena P., Aires-Mejia I. (2013). Detailed characterization of multiple myeloma circulating tumor cells shows unique phenotypic, cytogenetic, functional, and circadian distribution profile. Blood J. Am. Soc. Hematol..

[25] Dong L., Zhang Z., Smith K., Kuczler M., Reyes D., Amend S.R., Cho Y.-K., Xue W., Pienta K.J. (2020). The combination of size-based separation and selection-free technology provides higher circulating tumor cells detection sensitivity than either method alone in patients with metastatic prostate cancer. BJU Int..

[26] Riebensahm C., Joosse S.A., Mohme M., Hanssen A., Matschke J., Goy Y., Witzel I., Lamszus K., Kropidlowski J., Petersen C. (2019). Clonality of circulating tumor cells in breast cancer brain metastasis patients. Breast Cancer Res..

[27] Ye Z., Ding Y., Chen Z., Li Z., Ma S., Xu Z., Cheng L., Wang X., Zhang X., Ding N. (2019). Detecting and phenotyping of aneuploid circulating tumor cells in patients with various malignancies. Cancer Biol. Ther..

[28] Satelli A., Li S. (2011). Vimentin in cancer and its potential as a molecular target for cancer therapy. Cell. Mol. Life Sci..

[29] Coumans F.A.W., Doggen C.J.M., Attard G., De Bono J.S., Terstappen L.W.M.M. (2010). All circulating EpCAM+ CK+ CD45—Objects predict overall survival in castration-resistant prostate cancer. Ann. Oncol..

[30] Comaills V., Kabeche L., Morris R., Buisson R., Yu M., Madden M.W., LiCausi J.A., Boukhali M., Tajima K., Pan S. (2016). Genomic instability is induced by persistent proliferation of cells undergoing epithelial-to-mesenchymal transition. Cell Rep..

[31] Negrini S., Gorgoulis V.G., Halazonetis T.D. (2010). Genomic instability—An evolving hallmark of cancer. Nat. Rev. Mol. Cell Biol..

[32] Shen Z. (2011). Genomic instability and cancer: An introduction. J. Mol. Cell Biol..

[33] Meyn M.S. (1997). Chromosome instability syndromes: Lessons for carcinogenesis. Genetic Instability and Tumorigenesis.

[34] Geigl J.B., Obenauf A.C., Schwarzbraun T., Speicher M.R. (2008). Defining ‘chromosomal instability’. Trends Genet..

[35] Boveri T. (2008). Concerning the origin of malignant tumours by Theodor Boveri. Translated and annotated by Henry Harris. J. Cell Sci..

[36] Thompson S.L., Compton D.A. (2008). Examining the link between chromosomal instability and aneuploidy in human cells. J. Cell Biol..

[37] Bakhoum S.F., Compton D.A. (2012). Chromosomal instability and cancer: A complex relationship with therapeutic potential. J. Clin. Investig..

[38] Kobayashi K., Usami I., Kubota M., Nishio T., Kakazu N. (2005). Chromosome 7 abnormalities in acute megakaryoblastic leukemia associated with Down syndrome. Cancer Genet. Cytogenet..

[39] Sullivan S.G., Hussain R., Glasson E.J., Bittles A.H. (2007). The profile and incidence of cancer in Down syndrome. J. Intellect. Disabil. Res..

[40] Hunter A. (2003). High risk of malignancy in mosaic variegated aneuploidy syndrome. Am. J. Med Genet. Part A.

[41] Thompson S.L., Bakhoum S.F., Compton D.A. (2010). Mechanisms of chromosomal instability. Curr. Biol..

[42] Lengauer C., Kinzler K.W., Vogelstein B. (1997). Genetic instability in colorectal cancers. Nature.

[43] Yoon D.-S., Wersto R.P., Zhou W., Chrest F.J., Garrett E.S., Kwon T.K., Gabrielson E. (2002). Variable levels of chromosomal instability and mitotic spindle checkpoint defects in breast cancer. Am. J. Pathol..

[44] Haruki N., Harano T., Masuda A., Kiyono T., Takahashi T., Tatematsu Y., Shimizu S., Mitsudomi T., Konishi H., Osada H. (2001). Persistent increase in chromosome instability in lung cancer: Possible indirect involvement of p53 inactivation. Am. J. Pathol..

[45] Yuan B., Xu Y., Woo J.-H., Wang Y., Bae Y.K., Yoon D.-S., Wersto R.P., Tully E., Wilsbach K., Gabrielson E. (2006). Increased expression of mitotic checkpoint genes in breast cancer cells with chromosomal instability. Clin. Cancer Res..

[46] Ertych N., Stolz A., Stenzinger A., Weichert W., Kaulfuß S., Burfeind P., Aigner A., Wordeman L., Bastians H. (2014). Increased microtubule assembly rates influence chromosomal instability in colorectal cancer cells. Nat. Cell Biol..

[47] Bakhoum S.F., Genovese G., Compton D.A. (2009). Deviant kinetochore microtubule dynamics underlie chromosomal instability. Curr. Biol..

[48] Mowat M., Cheng A., Kimura N., Bernstein A., Benchimol S. (1985). Rearrangements of the cellular p53 gene in erythroleukaemic cells transformed by Friend virus. Nature.

[49] Hollstein M., Sidransky D., Vogelstein B., Harris C.C. (1991). p53 mutations in human cancers. Science.

[50] Donehower L.A. (1996). The p53-deficient mouse: A model for basic and applied cancer studies. Semin. Cancer Biol..

[51] Donehower L.A., Godley L.A., Aldaz C.M., Pyle R., Shi Y.-P., Pinkel D., Gray J., Bradley A., Medina D., Varmus H.E. (1996). The role of p53 loss in genomic instability and tumor progression in a murine mammary cancer model. Prog. Clin. Biol. Res..

[52] Fukasawa K., Wiener F., Woude G.F.V., Mai S. (1997). Genomic instability and apoptosis are frequent in p53 deficient young mice. Oncogene.

[53] Donehower L.A., Lozano G. (2009). 20 years studying p53 functions in genetically engineered mice. Nat. Rev. Cancer.

[54] Júnior G.B.C., Klumb C.E., Maia R.C. (2002). p53 e as hemopatias malignas. Rev. Bras. Cancerol..

[55] Kasiappan R., Shih H.-J., Chu K.-L., Chen W.-T., Liu H.-P., Huang S.-F., Choy C.O., Shu C.-L., Din R., Chu J.-S. (2009). Loss of p53 and MCT-1 overexpression synergistically promote chromosome instability and tumorigenicity. Mol. Cancer Res..

[56] Thompson S.L., Compton D.A. (2010). Proliferation of aneuploid human cells is limited by a p53-dependent mechanism. J. Cell Biol..

[57] Tighe A., Johnson V.L., Albertella M., Taylor S.S. (2001). Aneuploid colon cancer cells have a robust spindle checkpoint. EMBO Rep..

[58] Joukov V., Groen A.C., Prokhorova T., Gerson R., White E., Rodriguez A., Walter J.C., Livingston D.M. (2006). The BRCA1/BARD1 heterodimer modulates ran-dependent mitotic spindle assembly. Cell.

[59] Babu J.R., Jeganathan K.B., Baker D.J., Wu X., Kang-Decker N., Van Deursen J.M. (2003). Rae1 is an essential mitotic checkpoint regulator that cooperates with Bub3 to prevent chromosome missegregation. J. Cell Biol..

[60] Draviam V.M., Shapiro I., Aldridge B., Sorger P.K. (2006). Misorientation and reduced stretching of aligned sister kinetochores promote chromosome missegregation in EB1-or APC-depleted cells. EMBO J..

[61] Lepage C.C., Thompson L.L., Larson B., McManus K.J. (2020). An automated, single cell quantitative imaging microscopy approach to assess micronucleus formation, genotoxicity and chromosome instability. Cells.

[62] Kaldjian E.P., Ramirez A.B., Sun Y., Campton D.E., Werbin J.L., Varshavskaya P., Quarre S., George T., Madan A., Blau C.A. (2018). The RareCyte^®^ platform for next-generation analysis of circulating tumor cells. Cytom. Part A.

[63] Werbin J.L., Nordberg J.J., Tzucker J., Varshavskaya P., Stilwell J.L., Kaldjian E.P. (2017). RareCyte^®^ CTC analysis step 2: Detection of circulating tumor cells by CyteFinder^®^ automated scanning and semiautomated image analysis. Circulating Tumor Cells.

[64] Campton D.E., Ramirez A.B., Nordberg J.J., Drovetto N., Clein A.C., Varshavskaya P., Friemel B.H., Quarre S., Breman A., Dorschner M. (2015). High-recovery visual identification and single-cell retrieval of circulating tumor cells for genomic analysis using a dual-technology platform integrated with automated immunofluorescence staining. BMC Cancer.

[65] Schonhoft J.D., Zhao J.L., Jendrisak A., Carbone E.A., Barnett E.S., Hullings M.A., Gill A., Sutton R., Lee J., Dago A.E. (2020). Morphology-predicted large scale transition number in circulating tumor cells identifies a chromosomal instability biomarker associated with poor outcome in castration-resistant prostate cancer. Cancer Res..

[66] Jendrisak A., Lee J., Rodriguez A., Sutton R., Schonhoft J., Pramparo T., Wenstrup R., Wang Y. (2020). Abstract P4-01-03: Computer Vision and Machine Learning Allows for the Prediction of Genomic Instability Using Circulating Tumor Cell Morphology in Triple Negative Breast Cancer Patients.

[67] Marquard A.M., Eklund A.C., Joshi T., Krzystanek M., Favero F., Wang Z.C., Richardson A.L., Silver D.P., Szallasi Z., Birkbak N.J. (2015). Pan-cancer analysis of genomic scar signatures associated with homologous recombination deficiency suggests novel indications for existing cancer drugs. Biomark. Res..

[68] Kaldjian E., Drovetto N., Campton D., Ramirez A., Stilwell J., Clein A., Sabath D., Dumpit R., Nelson P. Multi-level analysis of circulating tumor cells in advanced prostate cancer using AccuCyte^®^–CyteFinder^®^. Proceedings of the 22nd Annual Prostate Cancer Foundation Scientific Retreat.

[69] Aguilar-Avelar C., Soto-García B., Aráiz-Hernández D., Yee-de León J.F., Esparza M., Chacón F., Delgado-Balderas J.R., Alvarez M.M., Trujillo-de Santiago G., Gómez-Guerra L.S. (2019). High-throughput automated microscopy of circulating tumor cells. Sci. Rep..

[70] Heidary M., Auer M., Ulz P., Heitzer E., Petru E., Gasch C., Riethdorf S., Mauermann O., Lafer I., Pristauz G. (2014). The dynamic range of circulating tumor DNA in metastatic breast cancer. Breast Cancer Res..

[71] Anantharaman A., Friedlander T., Lu D., Krupa R., Premasekharan G., Hough J., Edwards M., Paz R., Lindquist K., Graf R. (2016). Programmed death-ligand 1 (PD-L1) characterization of circulating tumor cells (CTCs) in muscle invasive and metastatic bladder cancer patients. BMC Cancer.

[72] Xu Y., Qin T., Li J., Wang X., Gao C., Xu C., Hao J., Liu J., Gao S., Ren H. (2017). Detection of circulating tumor cells using negative enrichment immunofluorescence and an in situ hybridization system in pancreatic cancer. Int. J. Mol. Sci..

[73] Liu H., Sun B., Wang S., Liu C., Lu Y., Li D., Liu X. (2017). Circulating tumor cells as a biomarker in pancreatic ductal adenocarcinoma. Cell. Physiol. Biochem..

[74] Qiu Z.-L., Wei W.-J., Sun Z.-K., Shen C.-T., Song H.-J., Zhang X.-Y., Zhang G.-Q., Chen X.-Y., Luo Q.-Y. (2018). Circulating tumor cells correlate with clinicopathological features and outcomes in differentiated thyroid cancer. Cell. Physiol. Biochem..

[75] Zhang J., Shi H., Jiang T., Liu Z., Lin P.P., Chen N. (2018). Circulating tumor cells with karyotyping as a novel biomarker for diagnosis and treatment of nasopharyngeal carcinoma. BMC Cancer.

[76] Chen Y., Yang Z., Wang Y., Wang J., Wang C. (2019). Karyotyping of circulating tumor cells for predicting chemotherapeutic sensitivity and efficacy in patients with esophageal cancer. BMC Cancer.

[77] Punnoose E.A., Atwal S., Liu W., Raja R., Fine B.M., Hughes B.G., Hicks R.J., Hampton G.M., Amler L.C., Pirzkall A. (2012). Evaluation of circulating tumor cells and circulating tumor DNA in non–small cell lung cancer: Association with clinical endpoints in a phase II clinical trial of pertuzumab and erlotinib. Clin. Cancer Res..

[78] Maheswaran S., Sequist L.V., Nagrath S., Ulkus L., Brannigan B., Collura C.V., Inserra E., Diederichs S., Iafrate A.J., Bell D.W. (2008). Detection of mutations in EGFR in circulating lung-cancer cells. N. Engl. J. Med..

[79] Pailler E., Adam J., Barthélémy A., Oulhen M., Auger N., Valent A., Borget I., Planchard D., Taylor M., André F. (2013). Detection of circulating tumor cells harboring a unique ALK rearrangement in ALK-positive non–small-cell lung cancer. J. Clin. Oncol..

[80] Provencio M., Pérez-Callejo D., Torrente M., Martin P., Calvo V., Gutiérrez L., Franco F., Coronado M.J., Cruz-Bermúdez J.L., Ruiz-Valdepeñas A.M. (2017). Concordance between circulating tumor cells and clinical status during follow-up in anaplastic lymphoma kinase (ALK) non-small-cell lung cancer patients. Oncotarget.

[81] Pailler E., Auger N., Lindsay C.R., Vielh P., Islas-Morris-Hernandez A., Borget I., Ngo-Camus M., Planchard D., Soria J.-C., Besse B. (2015). High level of chromosomal instability in circulating tumor cells of ROS1-rearranged non-small-cell lung cancer. Ann. Oncol..

[82] Pailler E., Oulhen M., Borget I., Remon J., Ross K., Auger N., Billiot F., Camus M.N., Commo F., Lindsay C.R. (2017). Circulating tumor cells with aberrant ALK copy number predict progression-free survival during Crizotinib treatment in ALK-rearranged non–small cell lung cancer patients. Cancer Res..

[83] Pawlikowska P., Faugeroux V., Oulhen M., Aberlenc A., Tayoun T., Pailler E., Farace F. (2019). Circulating tumor cells (CTCs) for the noninvasive monitoring and personalization of non-small cell lung cancer (NSCLC) therapies. J. Thorac. Dis..

[84] Carter L., Rothwell D.G., Mesquita B., Smowton C., Leong H.S., Fernandez-Gutierrez F., Li Y., Burt D.J., Antonello J., Morrow C.J. (2017). Molecular analysis of circulating tumor cells identifies distinct copy-number profiles in patients with chemosensitive and chemorefractory small-cell lung cancer. Nat. Med..

[85] Malihi P.D., Graf R.P., Rodriguez A., Ramesh N., Lee J., Sutton R., Jiles R., Velasco C.R., Sei E., Kolatkar A. (2020). Single-cell circulating tumor cell analysis reveals genomic instability as a distinctive feature of aggressive prostate cancer. Clin. Cancer Res..

[86] Gao Y., Ni X., Guo H., Su Z., Ba Y., Tong Z., Guo Z., Yao X., Chen X., Yin J. (2017). Single-cell sequencing deciphers a convergent evolution of copy number alterations from primary to circulating tumor cells. Genome Res..

[87] Lim S.B., Lim C.T., Lim W.-T. (2019). Single-Cell Analysis of Circulating Tumor Cells: Why Heterogeneity Matters. Cancers.

[88] Heitzer E., Auer M., Gasch C., Pichler M., Ulz P., Hoffmann E.M., Lax S., Waldispuehl-Geigl J., Mauermann O., Lackner C. (2013). Complex tumor genomes inferred from single circulating tumor cells by array-CGH and next-generation sequencing. Cancer Res..

[89] Lambros M.B., Seed G., Sumanasuriya S., Gil V., Crespo M., Fontes M., Chandler R., Mehra N., Fowler G., Ebbs B. (2018). Single-cell analyses of prostate cancer liquid biopsies acquired by apheresis. Clin. Cancer Res..

[90] Wang Y., Guo L., Feng L., Zhang W., Xiao T., Di X., Chen G., Zhang K. (2018). Single nucleotide variant profiles of viable single circulating tumour cells reveal CTC behaviours in breast cancer. Oncol. Rep..

[91] Kanwar N., Hu P., Bedard P., Clemons M., McCready D., Done S.J. (2015). Identification of genomic signatures in circulating tumor cells from breast cancer. Int. J. Cancer.

[92] Moyzis R.K., Buckingham J.M., Cram L.S., Dani M., Deaven L.L., Jones M.D., Meyne J., Ratliff R.L., Wu J.-R. (1988). A highly conserved repetitive DNA sequence,(TTAGGG) n, present at the telomeres of human chromosomes. Proc. Natl. Acad. Sci. USA.

[93] Chuang T.C.Y., Moshir S., Garini Y., Chuang A.Y.-C., Young I.T., Vermolen B., Van den Doel R., Mougey V., Perrin M., Braun M. (2004). The three-dimensional organization of telomeres in the nucleus of mammalian cells. BMC Biol..

[94] Vermolen B.J., Garini Y., Mai S., Mougey V., Fest T., Chuang T.-Y., Chuang A.-C., Wark L., Young I.T. (2005). Characterizing the three-dimensional organization of telomeres. Cytom. Part A.

[95] De Vos W.H., Hoebe R.A., Joss G.H., Haffmans W., Baatout S., Van Oostveldt P., Manders E.M. (2009). Controlled light exposure microscopy reveals dynamic telomere microterritories throughout the cell cycle. Cytom. Part A J. Int. Soc. Adv. Cytom..

[96] Louis S.F., Vermolen B.J., Garini Y., Young I.T., Guffei A., Lichtensztejn Z., Kuttler F., Chuang T.C., Moshir S., Mougey V. (2005). c-Myc induces chromosomal rearrangements through telomere and chromosome remodeling in the interphase nucleus. Proc. Natl. Acad. Sci. USA.

[97] Mai S., Garini Y. (2005). Oncogenic remodeling of the three-dimensional organization of the interphase nucleus: C-Myc induces telomeric aggregates whose formation precedes chromosomal rearrangements. Cell Cycle.

[98] Mai S., Garini Y. (2006). The significance of telomeric aggregates in the interphase nuclei of tumor cells. J. Cell. Biochem..

[99] Awe J.A., Xu M.C., Wechsler J., Benali-Furet N., Cayre Y.E., Saranchuk J., Drachenberg D., Mai S. (2013). Three-dimensional telomeric analysis of isolated circulating tumor cells (CTCs) defines CTC subpopulations. Transl. Oncol..

[100] Julius A.A., Yan A., Shah N., Ludger K., Kuzyk A., Xu M., Boles R., Saranchuk J., Drachenberg D., Mai S. (2014). 3D Nuclear Telomeric Signatures Define Circulating Tumor Cells (CTCs) and Characterize CTC Subpopulations in Intermediate Risk Prostate Cancer Patients.

[101] Drachenberg D., Awe J.A., Rangel Pozzo A., Saranchuk J., Mai S. (2019). Advancing Risk Assessment of Intermediate Risk Prostate Cancer Patients. Cancers.

[102] Wark L., Quon H., Ong A., Drachenberg D., Rangel-Pozzo A., Mai S. (2019). Long-Term Dynamics of Three Dimensional Telomere Profiles in Circulating Tumor Cells in High-Risk Prostate Cancer Patients Undergoing Androgen-Deprivation and Radiation Therapy. Cancers.

[103] Vishwakarma R., McManus K.J. (2020). Chromosome Instability; Implications in Cancer Development, Progression, and Clinical Outcomes. Cancers.

[104] Burrell R.A., McClelland S.E., Endesfelder D., Groth P., Weller M.-C., Shaikh N., Domingo E., Kanu N., Dewhurst S.M., Gronroos E. (2013). Replication stress links structural and numerical cancer chromosomal instability. Nature.

[105] Bakhoum S.F., Kabeche L., Murnane J.P., Zaki B.I., Compton D.A. (2014). DNA-damage response during mitosis induces whole-chromosome missegregation. Cancer Discov..

[106] Rowley J.D. (1973). A new consistent chromosomal abnormality in chronic myelogenous leukaemia identified by quinacrine fluorescence and Giemsa staining. Nature.

[107] Kalimutho M., Nones K., Srihari S., Duijf P.H., Waddell N., Khanna K.K. (2019). Patterns of genomic instability in breast cancer. Trends Pharmacol. Sci..

[108] O’Reilly E.A., Gubbins L., Sharma S., Tully R., Guang M.H.Z., Weiner-Gorzel K., McCaffrey J., Harrison M., Furlong F., Kell M. (2015). The fate of chemoresistance in triple negative breast cancer (TNBC). BBA Clin..

[109] Nedeljković M., Damjanović A. (2019). Mechanisms of Chemotherapy Resistance in Triple-Negative Breast Cancer—How We Can Rise to the Challenge. Cells.

[110] Ivanova E., Ward A., Wiegmans A.P., Richard D.J. (2020). Circulating Tumour Cells in Metastatic Breast Cancer: From genome instability to metastasis. Front. Mol. Biosci..

[111] Witzel I., Laakmann E., Weide R., Neunhöffer T., Park-Simon T.-J., Schmidt M., Fasching P.A., Hesse T., Polasik A., Mohrmann S. (2018). Treatment and outcomes of patients in the Brain Metastases in Breast Cancer Network Registry. Eur. J. Cancer.

[112] Powell E., Shao J., Yuan Y., Chen H.-C., Cai S., Echeverria G.V., Mistry N., Decker K.F., Schlosberg C., Do K.-A. (2016). p53 deficiency linked to B cell translocation gene 2 (BTG2) loss enhances metastatic potential by promoting tumor growth in primary and metastatic sites in patient-derived xenograft (PDX) models of triple-negative breast cancer. Breast Cancer Res..

[113] McDaniel A.S., Ferraldeschi R., Krupa R., Landers M., Graf R., Louw J., Jendrisak A., Bales N., Marrinucci D., Zafeiriou Z. (2017). Phenotypic diversity of circulating tumour cells in patients with metastatic castration-resistant prostate cancer. BJU Int..

[114] Morrison G.J., Goldkorn A. (2018). Development and application of liquid biopsies in metastatic prostate cancer. Curr. Oncol. Rep..

[115] Scher H.I., Graf R.P., Schreiber N.A., McLaughlin B., Jendrisak A., Wang Y., Lee J., Greene S., Krupa R., Lu D. (2017). Phenotypic heterogeneity of circulating tumor cells informs clinical decisions between AR signaling inhibitors and taxanes in metastatic prostate cancer. Cancer Res..

[116] Magbanua M.J.M., Sosa E.V., Scott J.H., Simko J., Collins C., Pinkel D., Ryan C.J., Park J.W. (2012). Isolation and genomic analysis of circulating tumor cells from castration resistant metastatic prostate cancer. BMC Cancer.

[117] Wyatt A.W., Annala M., Aggarwal R., Beja K., Feng F., Youngren J., Foye A., Lloyd P., Nykter M., Beer T.M. (2017). Concordance of circulating tumor DNA and matched metastatic tissue biopsy in prostate cancer. JNCI J. Natl. Cancer Inst..

[118] Antonarakis E.S., Lu C., Luber B., Wang H., Chen Y., Nakazawa M., Nadal R., Paller C.J., Denmeade S.R., Carducci M.A. (2015). Androgen receptor splice variant 7 and efficacy of taxane chemotherapy in patients with metastatic castration-resistant prostate cancer. JAMA Oncol..

[119] Losi L., Baisse B., Bouzourene H., Benhattar J. (2005). Evolution of intratumoral genetic heterogeneity during colorectal cancer progression. Carcinogenesis.

[120] Almendro V., Marusyk A., Polyak K. (2013). Cellular heterogeneity and molecular evolution in cancer. Annu. Rev. Pathol. Mech. Dis..

[121] Gerlinger M., Horswell S., Larkin J., Rowan A.J., Salm M.P., Varela I., Fisher R., McGranahan N., Matthews N., Santos C.R. (2014). Genomic architecture and evolution of clear cell renal cell carcinomas defined by multiregion sequencing. Nat. Genet..

[122] Colombino M., Capone M., Lissia A., Cossu A., Rubino C., De Giorgi V., Massi D., Fonsatti E., Staibano S., Nappi O. (2012). BRAF/NRAS mutation frequencies among primary tumors and metastases in patients with melanoma. J. Clin. Oncol..

[123] Mao C., Wu X.-Y., Yang Z.-Y., Threapleton D.E., Yuan J.-Q., Yu Y.-Y., Tang J.-L. (2015). Concordant analysis of KRAS, BRAF, PIK3CA mutations and PTEN expression between primary colorectal cancer and matched metastases. Sci. Rep..

[124] Gerlinger M., Rowan A.J., Horswell S., Larkin J., Endesfelder D., Gronroos E., Martinez P., Matthews N., Stewart A., Tarpey P. (2012). Intratumor heterogeneity and branched evolution revealed by multiregion sequencing. N. Engl. J. Med..

[125] Wang Y., Waters J., Leung M.L., Unruh A., Roh W., Shi X., Chen K., Scheet P., Vattathil S., Liang H. (2014). Clonal evolution in breast cancer revealed by single nucleus genome sequencing. Nature.

[126] Jamal-Hanjani M., Quezada S.A., Larkin J., Swanton C. (2015). Translational implications of tumor heterogeneity. Clin. Cancer Res..

[127] Huntly B.J., Gilliland D.G. (2005). Summing up cancer stem cells. Nature.

[128] Eaves C.J. (2008). Here, there, everywhere?. Nature.

[129] Merlo L.M., Pepper J.W., Reid B.J., Maley C.C. (2006). Cancer as an evolutionary and ecological process. Nat. Rev. Cancer.

[130] Navin N., Hicks J. (2011). Future medical applications of single-cell sequencing in cancer. Genome Med..

[131] Mohamed Suhaimi N.-A., Foong Y.M., Lee D.Y.S., Phyo W.M., Cima I., Lee E.X.W., Goh W.L., Lim W.-Y., Chia K.S., Kong S.L. (2015). Non-invasive sensitive detection of KRAS and BRAF mutation in circulating tumor cells of colorectal cancer patients. Mol. Oncol..

[132] Mostert B., Jiang Y., Sieuwerts A.M., Wang H., Bolt-de Vries J., Biermann K., Kraan J., Lalmahomed Z., Van Galen A., De Weerd V. (2013). KRAS and BRAF mutation status in circulating colorectal tumor cells and their correlation with primary and metastatic tumor tissue. Int. J. Cancer.

[133] Lyberopoulou A., Aravantinos G., Efstathopoulos E.P., Nikiteas N., Bouziotis P., Isaakidou A., Papalois A., Marinos E., Gazouli M. (2015). Mutational analysis of circulating tumor cells from colorectal cancer patients and correlation with primary tumor tissue. PLoS ONE.

[134] Fabbri F., Carloni S., Zoli W., Ulivi P., Gallerani G., Fici P., Chiadini E., Passardi A., Frassineti G.L., Ragazzini A. (2013). Detection and recovery of circulating colon cancer cells using a dielectrophoresis-based device: KRAS mutation status in pure CTCs. Cancer Lett..

[135] Kalikaki A., Politaki H., Souglakos J., Apostolaki S., Papadimitraki E., Georgoulia N., Tzardi M., Mavroudis D., Georgoulias V., Voutsina A. (2014). KRAS genotypic changes of circulating tumor cells during treatment of patients with metastatic colorectal cancer. PLoS ONE.

[136] Pestrin M., Bessi S., Galardi F., Truglia M., Biggeri A., Biagioni C., Cappadona S., Biganzoli L., Giannini A., Di Leo A. (2009). Correlation of HER2 status between primary tumors and corresponding circulating tumor cells in advanced breast cancer patients. Breast Cancer Res. Treat..

[137] Aktas B., Müller V., Tewes M., Zeitz J., Kasimir-Bauer S., Loehberg C.R., Rack B., Schneeweiss A., Fehm T. (2011). Comparison of estrogen and progesterone receptor status of circulating tumor cells and the primary tumor in metastatic breast cancer patients. Gynecol. Oncol..

[138] Ligthart S.T., Bidard F.-C., Decraene C., Bachelot T., Delaloge S., Brain E., Campone M., Viens P., Pierga J.-Y., Terstappen L. (2013). Unbiased quantitative assessment of Her-2 expression of circulating tumor cells in patients with metastatic and non-metastatic breast cancer. Ann. Oncol..

[139] Fisher R., Pusztai L., Swanton C. (2013). Cancer heterogeneity: Implications for targeted therapeutics. Br. J. Cancer.

[140] Rangel-Pozzo A., Liu S., Wajnberg G., Wang X., Ouellette R.J., Hicks G.G., Drachenberg D., Mai S. (2020). Genomic Analysis of Localized High-Risk Prostate Cancer Circulating Tumor Cells at the Single-Cell Level. Cells.

[141] Lowes L.E., Bratman S.V., Dittamore R., Done S., Kelley S.O., Mai S., Morin R.D., Wyatt A.W., Allan A.L. (2016). Circulating tumor cells (CTC) and cell-free DNA (cfDNA) workshop 2016: Scientific opportunities and logistics for cancer clinical trial incorporation. Int. J. Mol. Sci..

[142] Scher H.I., Graf R., Schreiber N.A., McLaughlin B., Lu D., Louw J., Jendrisak A., Greene S., Rodriguez A., Dugan L. (2016). AR-V7 and CTC Heterogeneity Biomarkers Additively to Predict Patient (pt) Outcomes with Taxanes Relative to Approved AR Targeted Therapy. JCO.

[143] Scher H.I., Jendrisak A., Graf R., Schreiber N.A., McLaughlin B., Greene S., Rodriguez A., Louw J., Dugan L., Leitz L. (2016). CTC Phenotype Classifier to Identify mCRPC Patients (pts) with High Genomic Instability CTCs and to Predict Failure of Androgen Ecreptor Signaling (AR Tx) and Taxane (T) Systemic Therapies. JCO.

[144] Osborne C.K., Schiff R. (2011). Mechanisms of endocrine resistance in breast cancer. Annu. Rev. Med..

[145] Su K.-Y., Chen H.-Y., Li K.-C., Kuo M.-L., Yang J.C., Chan W.-K., Ho B.-C., Chang G.-C., Shih J.-Y., Yu S.-L. (2012). Pretreatment epidermal growth factor receptor (EGFR) T790M mutation predicts shorter EGFR tyrosine kinase inhibitor response duration in patients with non-small-cell lung cancer. J. Clin. Oncol..

[146] Kwak E.L., Sordella R., Bell D.W., Godin-Heymann N., Okimoto R.A., Brannigan B.W., Harris P.L., Driscoll D.R., Fidias P., Lynch T.J. (2005). Irreversible inhibitors of the EGF receptor may circumvent acquired resistance to gefitinib. Proc. Natl Acad. Sci. USA.

[147] Kulasinghe A., Lim Y., Kapeleris J., Warkiani M., O’Byrne K., Punyadeera C. (2020). The Use of Three-Dimensional DNA Fluorescent in Situ Hybridization (3D DNA FISH) for the Detection of Anaplastic Lymphoma Kinase (ALK) in Non-Small Cell Lung Cancer (NSCLC) Circulating Tumor Cells. Cells.

[148] Pailler E., Faugeroux V., Oulhen M., Mezquita L., Laporte M., Honoré A., Lecluse Y., Queffelec P., NgoCamus M., Nicotra C. (2019). Acquired resistance mutations to ALK inhibitors identified by single circulating tumor cell sequencing in ALK-Rearranged non–Small-Cell Lung Cancer. Clin. Cancer Res..

[149] Antonarakis E.S., Lu C., Wang H., Luber B., Nakazawa M., Roeser J.C., Chen Y., Mohammad T.A., Chen Y., Fedor H.L. (2014). AR-V7 and resistance to enzalutamide and abiraterone in prostate cancer. N. Engl. J. Med..

[150] Diamantopoulou Z., Castro-Giner F., Aceto N. (2020). Circulating tumor cells: Ready for translation?. J. Exp. Med..

[151] Gkountela S., Castro-Giner F., Szczerba B.M., Vetter M., Landin J., Scherrer R., Krol I., Scheidmann M.C., Beisel C., Stirnimann C.U. (2019). Circulating tumor cell clustering shapes DNA methylation to enable metastasis seeding. Cell.

[152] Kim C., Gao R., Sei E., Brandt R., Hartman J., Hatschek T., Crosetto N., Foukakis T., Navin N.E. (2018). Chemoresistance evolution in triple-negative breast cancer delineated by single-cell sequencing. Cell.

